# Circadian disruption of core body temperature in trauma patients: a single-center retrospective observational study

**DOI:** 10.1186/s40560-019-0425-x

**Published:** 2020-01-06

**Authors:** Aurélien Culver, Benjamin Coiffard, François Antonini, Gary Duclos, Emmanuelle Hammad, Coralie Vigne, Jean-Louis Mege, Karine Baumstarck, Mohamed Boucekine, Laurent Zieleskiewicz, Marc Leone

**Affiliations:** 1 Service d’Anesthésie et de Réanimation, APHM, Hôpital Nord, Aix Marseille Université, Chemin des Bourrely, 13915 Marseille, France; 2 Médecine Intensive – Réanimation, APHM, Hôpital Nord, Aix Marseille Université, Marseille, France; 3 CNRS, IRD, MEPHI, IHU Méditerranée Infection, Aix Marseille Université, Marseille, France; 4 APHM, EA 3279 CEReSS, School of Medicine - La Timone Medical Campus, Health Service Research and Quality of Life Center, Aix Marseille Université, Marseille, France

**Keywords:** Critical care, Multiple trauma, Mortality, Body temperature, Circadian rhythm, Mathematical computing

## Abstract

**Background:**

Circadian clock alterations were poorly reported in trauma patients, although they have a critical role in human physiology. Core body temperature is a clinical variable regulated by the circadian clock. Our objective was to identify the circadian temperature disruption in trauma patients and to determine whether these disruptions were associated with the 28-day mortality rate.

**Methods:**

A retrospective and observational single-center cohort study was conducted. All adult severe trauma patients admitted to the intensive care unit of Aix Marseille University, North Hospital, from November 2013 to February 2018, were evaluated. The variations of core body temperature for each patient were analyzed between days 2 and 3 after intensive care unit admission. Core body temperature variations were defined by three parameters: mesor, amplitude, and period. A logistic regression model was used to determine the variables influencing these three parameters. A survival analysis was performed assessing the association between core body temperature rhythm disruption and 28-day mortality rate. A post hoc subgroup analysis focused on the patients with head trauma.

**Results:**

Among the 1584 screened patients, 248 were included in this study. The period differed from 24 h in 177 (71%) patients. The mesor value (°C) was associated with body mass index and ketamine use. Amplitude (°C) was associated with ketamine use only. The 28-day mortality rate was 18%. For all trauma patients, age, body mass index, intracranial hypertension, and amplitude were independent risk factors. The patients with a mesor value < 36.9 °C (*p* < 0.001) and an amplitude > 0.6 °C (*p* < 0.001) had a higher 28-day mortality rate. Among the patients with head trauma, mesor and amplitude were identified as independent risk factors (HR = 0.40, 95% CI [0.23–0.70], *p* = 0.001 and HR = 4.73, 95% CI [1.38–16.22], *p* = 0.01).

**Conclusions:**

Our results highlight an association between core body temperature circadian alteration and 28-day mortality rate. This association was more pronounced in the head trauma patients than in the non-head trauma patients. Further studies are needed to show a causal link and consider possible interventions.

## Background

Major trauma is the leading cause of mortality among patients admitted in the intensive care unit (ICU) [1]. Two components affect the outcome of trauma patients: organ damage due to direct trauma and post-traumatic systemic inflammation progressing to multiorgan failure [2]. These pathologic events are responsible for disrupting the physiological mechanisms.

The circadian clock is a biological function that has been particularly preserved throughout species evolution, and it is present in almost all living organisms [3]. In humans, circadian clock is regulated by a central stimulator located in the suprachiasmatic nuclei of the hypothalamus [4, 5]. It synchronizes intracellular processes and vital functions, including core body temperature (CBT), sleep, cardiovascular and respiratory functions, coagulation, and immunity to the external environment [6, 7]. This system is directed by a set of specific genes known as the “clock genes,” of which about 10 are identified [8, 9].

CBT is a stable physiological variable, whose variations are correlated to the activity of the circadian clock [10, 11]. In healthy subjects, the normal values oscillate by about 0.5 °C per day and are around an average of 37.0 °C [12]. CBT reaches its lowest value between 4:00 am and 6:00 am and rises gradually during the day to a maximal value between 4:00 pm and 6:00 pm [13]. In routine, clinicians interpret its value dichotomously, reporting the presence or absence of fever. However, several studies have reported the importance of considering temperature variations rather than a single isolated value [14, 15].

The circadian clock cannot be directly evaluated in humans. Three variables have been used to indirectly assess the circadian clock: CBT and the levels of cortisol and melatonin [16, 17]. Studies have assessed the circadian clock of trauma patients admitted to the ICU [16–18]. Circadian clock disruption seems to be frequent in this population [16–21]. In a previous study, we showed that trauma patients exhibited early impaired circadian rhythms of biological mediators [22]. Importantly, this early disruption was associated with an increased risk of sepsis development during the ICU stay.

Here, we hypothesized that the assessment of CBT variations is relevant, particularly at the early phase of ICU admission, to identify the patients at high risk of mortality. Our study aims at identifying an association between circadian clock disruption based on CBT analysis and 28-day mortality rate in trauma patients.

## Materials and methods

### Study design

A single-center retrospective observational study was conducted in the Trauma Center of Aix Marseille University, Marseille, France. This center includes a 15-bed ICU and a shock room dedicated to the early management of trauma patients. As per the French law [23], the research protocol has been approved by the Ethics Committee of the French Society of Anesthesia and Intensive Care Medicine (IRB CERAR-00010254) and by the local Computer and Freedom Committee (CIL 2018-35). As the study was retrospective, the written consent was waived by our Ethics Committee.

### Patients

All patients registered in our department’s database and admitted to ICU for major trauma between November 2013 and February 2018 and monitored for at least 72 h of continuous CBT measurements were included. Major trauma was defined as a patient with at least one life-threatening lesion or whose trauma severity suggests the existence of such a lesion. Vittel algorithm was used to define a trauma patient [24]. Exclusion criteria were as follows: (1) death within the first 72 h; (2) ICU stay < 72 h; (3) absence of CBT monitoring during the first 72 h after ICU admission; (4) incomplete data on CBT monitoring with a lack of data exceeding a total of 6 h between 24 h and 72 h after ICU admission; and (5) use of a targeted temperature control system and renal replacement therapy. Because targeted temperature control was used for patients with cardiac arrest, they were excluded from the study.

### Demographic data

Demographic and clinical data were retrospectively extracted from the electronic medical files. At admission, we recorded age, gender, body mass index (BMI), Glasgow Coma Scale score, Simplified Acute Physiology Score (SAPS2), Acute Injury Score (AIS), and Injury Severity Score (ISS).

During the first 72 h, we collected data on the intracranial hypertension (defined as an episode of intracranial pressure > 20 mmHg for more than 5 min), mechanical ventilation, need for urgent surgery or craniectomy, administration of acetaminophen, use of sedative agents (benzodiazepine, ketamine, sufentanil, propofol), use of neuromuscular blocker agents, intra-hospital transport, and blood transfusion. The durations of mechanical ventilation, catecholamine infusion (if any), ICU, and hospital stay and the 28-day mortality rate were also recorded.

### Core body temperature curves

For each patient, the first 72 h of CBT measurements were extracted from our ICU database. Since the trauma patients were often disconnected from the monitors for radiological or surgical procedures during the first 24 h, we selected the timeslot between 24 h and 72 h after ICU admission. CBT was measured using a Foley urinary catheter (400 TM temperature sensor, Covidien™, Boulder, USA) directly connected to the patient’s monitor (Intellivue 70, Philips, Eindhoven, Netherlands). CBT data were recorded at the rate of 1 value per minute (representing 2880 points for the study period).

### Patient management

The management of trauma patients was based on European guidelines [25]. The trauma patients were bedridden for the first few ICU days, and they were fed by the enteral route continuously if possible. According to our protocol based on international guidelines [25], sedation was used to target a Richmond Agitation-Sedation Scale (RASS) at 0 in the absence of intracranial hypertension or acute respiratory distress syndrome (RASS at − 5). The ambient ICU temperature was automatically set between 22 °C and 24 °C through a thermostat. Light was set as daylight during the day and dark for night. Acetaminophen was administered in the patients with brain injury and body temperature exceeding 37.5 °C and those with pain above 30 in the visual analog scale or above 6 in the behavioral pain scale. Only six patients received additive use of non-steroidal anti-inflammatory drugs. Nursing care was provided continuously [26]. Intensivists were in a house for 24 h/7 days with rounds at 11 am, 6 pm, and 11 pm. Our ICU follows the organization of French ICUs [26].

### Fourier transformation and Cosinor analysis

We assessed the CBT for each patient. To determine the most probable time of temporal oscillation (first harmonic), a spectral resolution method (Fourier transformation) was used [27]. Using the least squares method, unique Cosinor analysis then adjusted the sinusoidal wave to the time series (Additional file 1). This model uses the following formula: *y* = mes + amp × cos (2(*t* − ϕ)/*p*), where “*p*” is the period found by the Fourier transformation, “*y*” is the marker value (CBT), “*t*” represents the time of day in decimal mode, “mes,” “amp,” and “ϕ” represent respectively the mesor, the amplitude, and the acrophase of the rhythm (see Additional file 2 and Additional file 3 for the definition of these variables). Additional file 4 illustrates four examples of CBT rhythm modeling.

### Statistical analysis

Statistical analyses were performed with the public software R version 3.5.1 R Development Core Team (2005) (R: A language and environment for statistical computing. R Foundation for Statistical Computing, Vienna, Austria. ISBN 3-900051-07-0, URL http://www.R-project.org). The initial population characteristics were expressed in number (percentages) for categorical variables, and as the mean ± standard deviation (SD) or median and interquartile [IQR] for continuous data according to the distribution (Shapiro-Wilk test). Associations between clinical variables and circadian parameters (period, mesor, and amplitude) were studied by logistic regression with the general linear model. Regression coefficients ß ± standard error (SE) and odds ratios (OR) obtained in this way were used to assess the relative predictive effects of the independent variables. A survival analysis was performed using Cox’s proportional risk model to determine the factors associated with 28-day mortality. Relative risks, hazard ratio (HR), and their 95% confidence intervals (95% CI) were calculated. All variables with *p* < 0.05 values in univariate models were included in the final multivariate models. The 28-day survival between groups was calculated by the log-rank test, and survival curves were obtained by the Kaplan-Meier method. To form groups with the highest survival difference, the best significant threshold of the circadian parameter for predicting 28-day mortality was calculated with the *cutp* function of R to determine the optimal cut point for a continuous variable in the Cox model. A post hoc subgroup analysis was performed in the patients with head trauma defined by the notification of this term in the initial medical file. For all tests, statistical significance was defined by *p* values less than 0.05.

## Results

### Patients

**Fig. 1:**
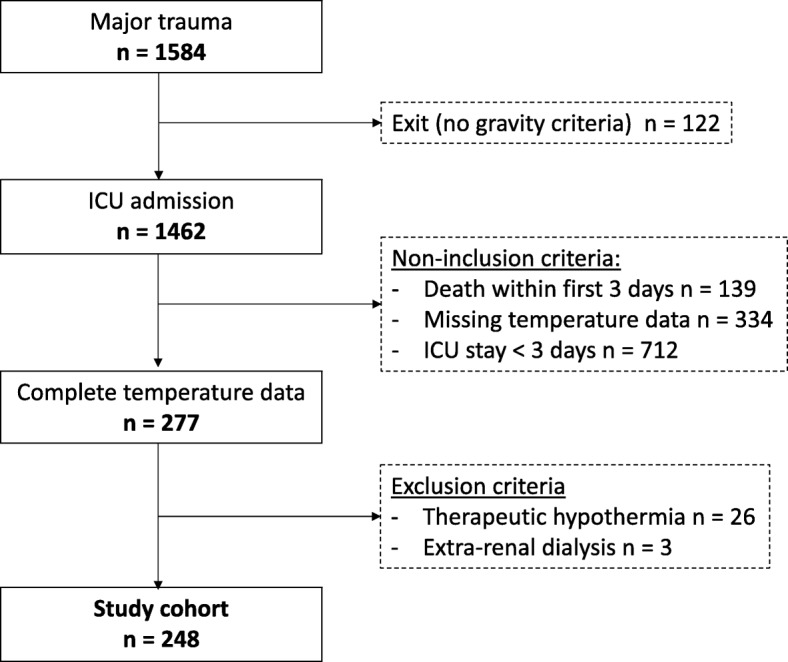
Population flowchart

A total of 1584 patients were screened during the 4-year study period. Among them, 248 (15.7%) fulfilled the inclusion criteria (Fig. 1). The clinical features and initial treatments are reported in Table 1. Most patients were young males. Mechanical ventilation and urgent surgery were required in 74% and 57% of the patients, respectively. The 28-day mortality rate was 18%.

**Table 1:** Patient characteristics and outcome

Variables	Cohort (*n* = 248)
Age, years	36 [23–53]
Sex, men	181 (73.0)
BMI, kg/m^2^	24 [22–26]
Initial trauma condition	
Glasgow Coma Scale	8 [5–14]
Head injury	176 (71.0)
Intracranial hypertension	80 (32.3)
Surgery in the first 24 h	142 (57.3)
SAPS2	47 ± 16
Severity of trauma	
ISS	34 [25–43]
AIS head or neck	4.5 [0–5]
AIS chest	3 [0–3]
AIS abdomen or pelvic	0 [0–2]
AIS extremities	0 [0–2]
AIS rachis	0 [0–2]
Treatments	
Mechanical ventilation	184 (74.2)
Acetaminophen	159 (64.1)
Benzodiazepine	136 (54.8)
Opioids	202 (81.4)
Neuro-muscular blockers	37 (14.9)
Ketamine	24 (9.7)
Craniectomy	13 (5.2)
Transfusion	0 [0–3]
In-hospital transport	135 (54.4)
Outcome	
28-day mortality	45 (18.1)
Days on mechanical ventilation	6 [3–13]
Days on vasopressor infusion	2 [1–4]
Day in the ICU stay	9 [5–18]
Days in the hospital	12 [7–23]

Results are expressed as median [IQR] for quantitative variables and number (%) for categorical variables

*BMI* body mass index, *SAPS2* Severity Assessment Physiology Score 2, *ISS* Injury Severity Score, *AIS* Abbreviated Injury Scale

### Core body temperature rhythm

**Fig. 2:**
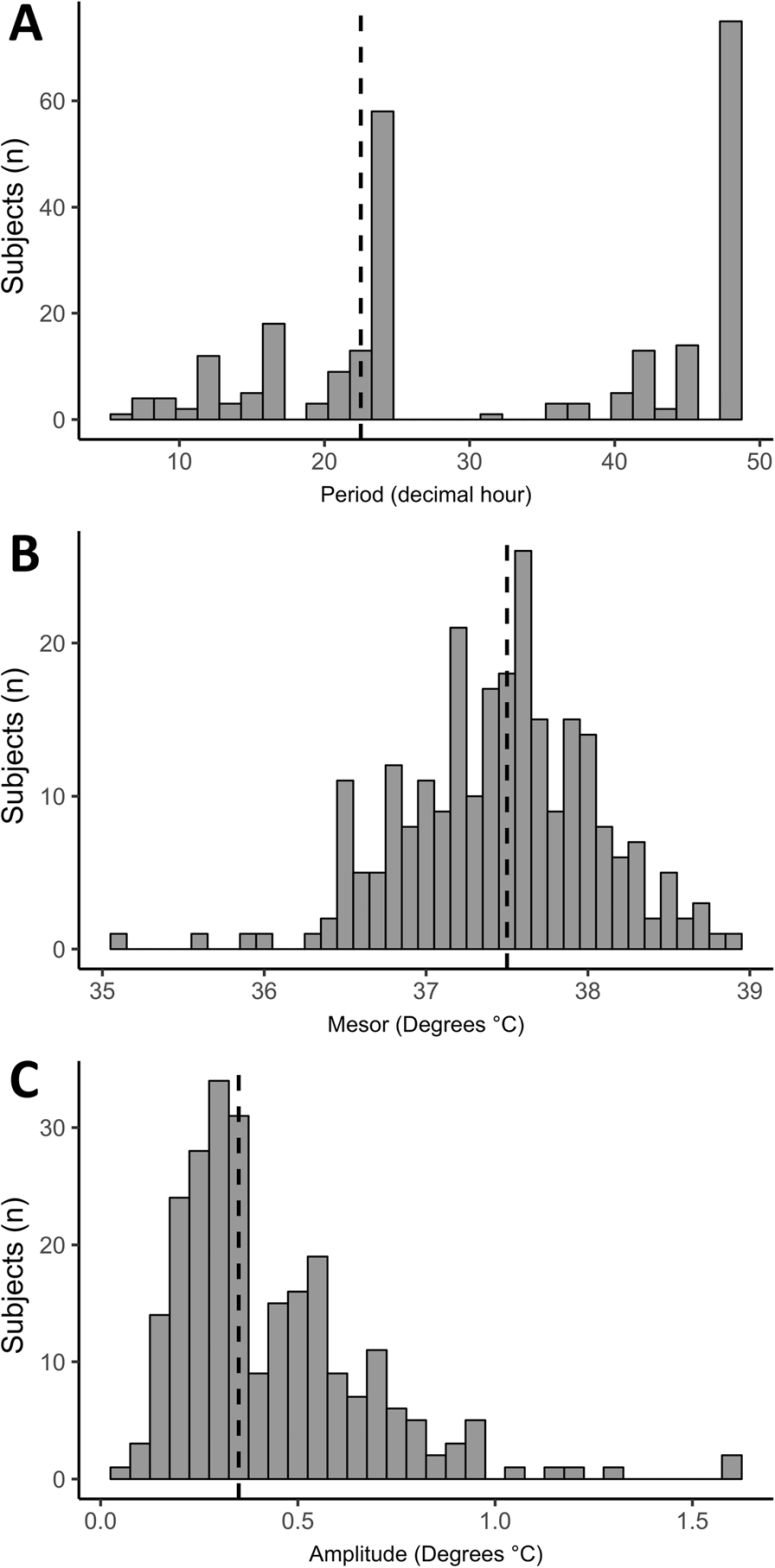
Distribution of the three rhythm parameters of the temperature. **a** Period. **b** Mesor. **c** Amplitude. The median [IQR] values (discontinuous bar) of the period, mesor, and amplitude were 24 h [23–48], 37.5 °C [37.1–37.9], 0.35 °C [0.26–0.54], respectively

Periodic regressions were extracted from individual data (Additional file 5). Figure 2 shows the distribution of the three circadian parameters (i.e., period, mesor, and amplitude). The median [IQR] values of the mesor, amplitude, and period were 37.5 °C [37.1–37.9], 0.35 °C [0.26–0.54], and 24 h [23–48], respectively. Only 71 (29%) patients had a period between 22 h and 26 h, while 75 (30%) patients exhibited a period longer than or equal to 48 h. In contrast, the mesor and amplitude were in the normal ranges.

### Patient characteristics associated with core body temperature rhythm

The results of the univariate logistic regression analysis are reported in Additional file 6. In the multivariate analysis, the mesor of the CBT significantly increased with increasing BMI while it decreased with ketamine infusion (Additional file 7). The amplitude significantly increased with ketamine infusion.

### Core body temperature rhythm and 28-day mortality

The results of the univariate and multivariate Cox proportional survival analyses are reported in Table 2. In the multivariate analysis, age, intracranial hypertension, and amplitude of the CBT were identified as independent risk factors for 28-day mortality rate. The mesor (as a continuous variable) was almost significantly associated with 28-day mortality rate (HR = 0.62, 95% CI [0.37–1.03], *p* = 0.064). BMI was a protective factor of 28-day mortality.

**Table 2:** Survival analysis assessing 28-day mortality, patient characteristics, and circadian rhythm parameters of the temperature

	Univariate		Multivariate	
	HR [95% CI]	*p*	HR [95% CI]	*p*
Clinical variables				
Age	1.04 [1.03–1.06]	< 0.001	1.06 [1.04–1.08]	< 0.001
Sex (male)	1.73 [0.81–3.72]	0.16		
Body mass index	0.91 [0.83–0.99]	0.04	0.83 (0.75–0.93)	< 0.001
Glasgow Coma Scale	0.90 [0.84–0.97]	0.005		
Traumatic brain injury	2.05 [0.95–4.39]	0.07		
Intracranial hypertension^a^	5.70 [3.03–10.73]	< 0.001	5.60 [2.91–10.76]	< 0.001
Surgery at admission	0.74 [0.41–1.33]	0.32		
ISS	1.01 [0.99–1.03]	0.26		
Temperature rhythm				
Period	1.00 [0.98–1.02]	0.65		
Mesor	0.42 [0.27–0.66]	< 0.001	0.62 [0.37–1.03]	0.064
Amplitude	4.25 [1.58–11.41]	0.004	3.28 [1.15–9.30]	0.026

The analyses were performed using Cox regression model to estimate hazard ratios (HR) and their 95% confidence intervals (95% CIs)

^a^Glasgow Coma Scale score not included in the multivariate analysis owing to collinearity with intracranial hypertension

**Fig. 3:**
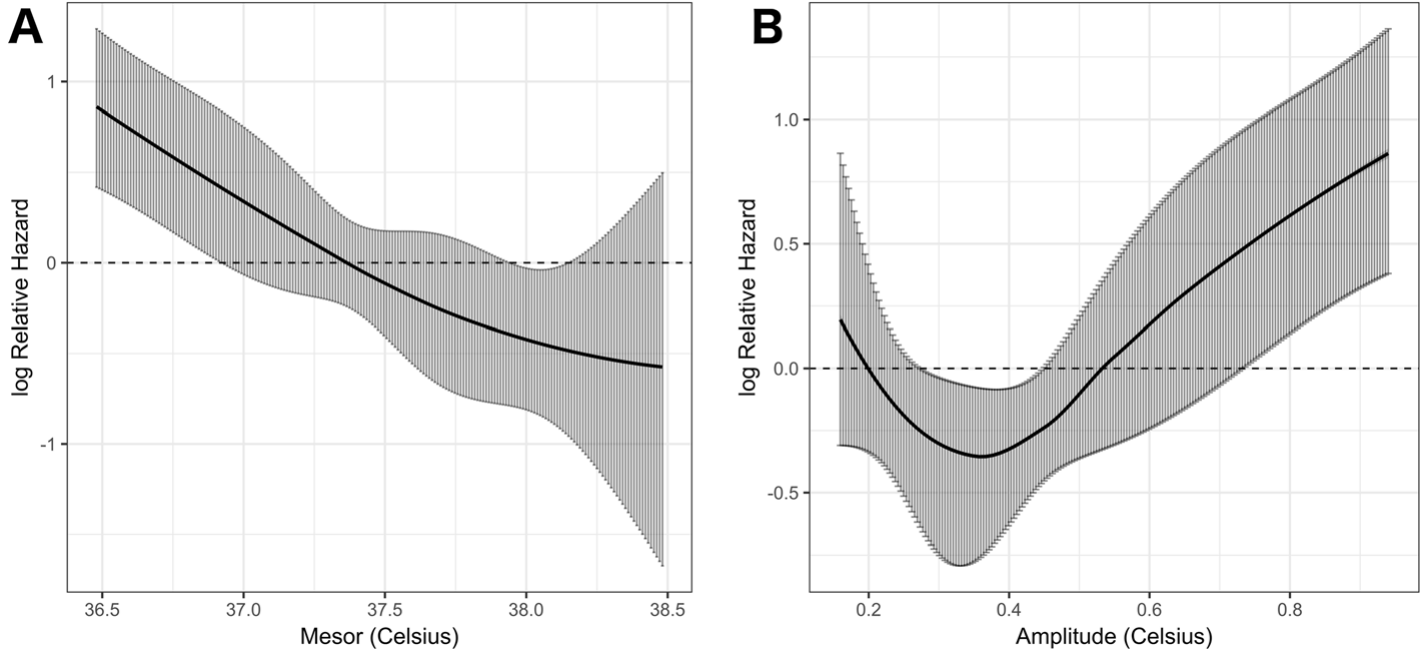
Cox regression model fitting the association between temperature and the log relative hazard of 28-day mortality. **a** Mesor. **b** Amplitude. Black curve represents the estimates of the model and gray bars represent the 95% confidence interval bands

Figure 3 shows the Cox regression model fitting the continuous association between mesor or amplitude and the log relative hazard of 28-day mortality. The risk of 28-day mortality was increased for the lowest mesor (< 37.5 °C) and highest amplitude (> 0.5 °C) values. A protective effect was identified in patients with CBT amplitude close to the physiological values (between 0.2 and 0.5 °C).

**Fig. 4:**
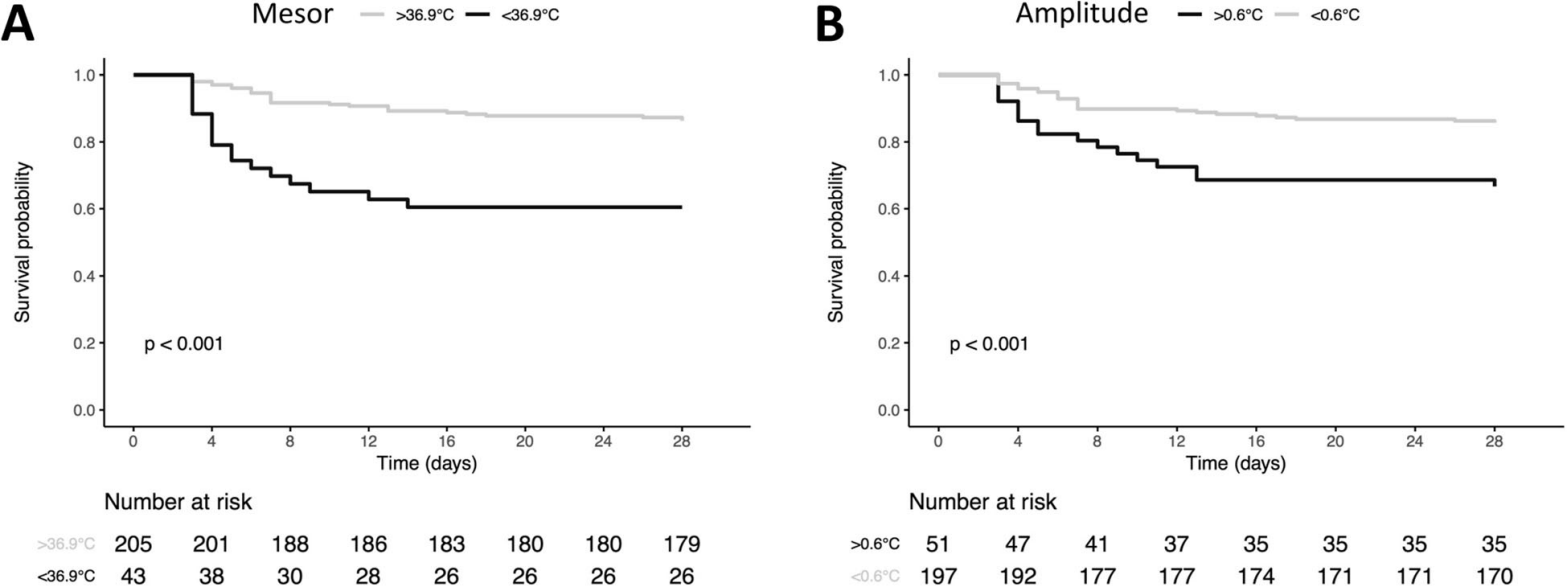
Kaplan-Meier curves representing the 28-day ICU survival according to the most significant threshold of mesor (**a**) and amplitude (**b**). *p* values result from log-rank analysis between groups

The best significant thresholds of the circadian parameters for predicting 28-day mortality were 36.9 °C for the mesor (*p* < 0.001) and 0.6 °C for the amplitude (*p* < 0.001) (see Kaplan-Meier curves on Fig. 4).

### Head trauma patients

**Fig. 5:**
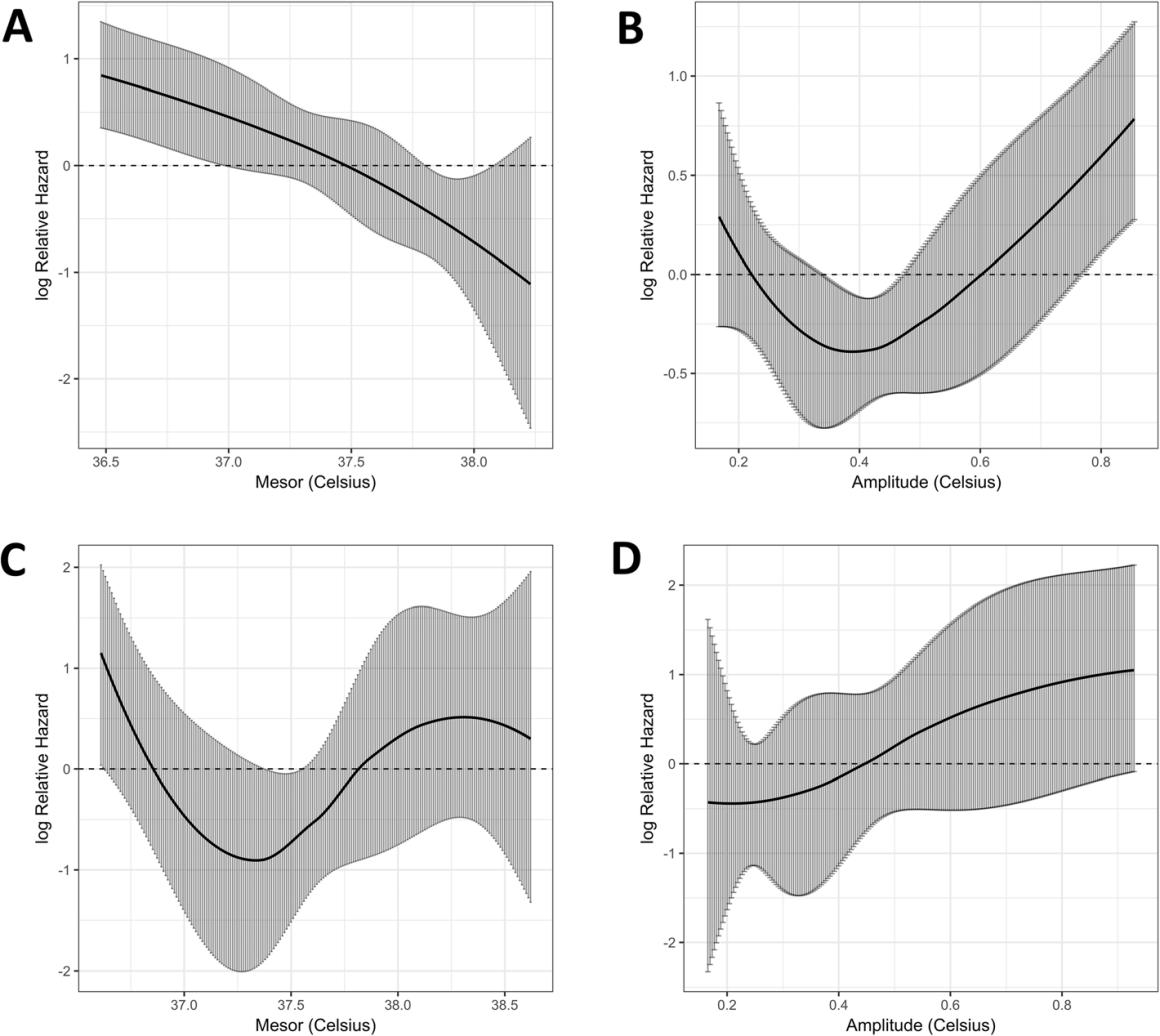
Cox regression model fitting the association between temperature and the log relative hazard of 28-day mortality according to head trauma. **a** Mesor in head trauma group. **b** Amplitude in head trauma group. **c** Mesor in non-head trauma group. **d** Amplitude in non-head trauma group. Black curve represents the estimates of the model and gray bars represent the 95% confidence interval bands

The 176 head trauma patients were compared with the non-head trauma patients (Table 3). The ISS and the exposure to mechanical ventilation were higher in the head trauma patients, as compared with the non-head trauma patients. The results of the univariate and multivariate analyses in the head trauma and the non-head trauma patients are reported in Table 4. Mesor and amplitude were identified as independent risk factors in the head trauma patients (HR = 0.40, 95% CI [0.23–0.70], *p* = 0.001 and HR = 4.73, 95% CI [1.38–16.22], *p* = 0.01). None of the three variables were independently associated with 28-day mortality in the non-head trauma patients. Figure 5 illustrates the Cox regression model fitting the continuous association between mesor or amplitude and the log relative hazard of 28-day mortality for head trauma patients and non-head trauma patients.

**Table 3:** Patient characteristics and outcome according to head trauma

Variables	Head trauma (*n* = 176)	Non-head trauma (*n* = 72)	*p*
Age, years	36 [23–54]	35 [24–49]	0.86
Sex, men	127 (72.0)	54 (75.0)	0.65
BMI, kg/m^2^	24 [22–26]	24 [23–26]	0.36
Initial trauma condition			
Glasgow Coma Scale	6 [4–10]	15 [14–15]	< 0.001
Head injury			
Intracranial hypertension	80 (45.5)	0 (0.0)	–
Surgery in the first 24 h	84 (47.7)	58 (80.6)	< 0.001
SAPS2	48 ± 16	42 ± 17	< 0.001
Severity of the trauma			
ISS	34 [25–43]	27 [23–41]	0.02
AIS head or neck	5 [4–5]	0 [0–0]	–
AIS chest	2 [0–3]	3 [0–3]	0.02
AIS abdomen or pelvic	0 [0–0]	2 [0–4]	< 0.001
AIS extremities	0 [0–2]	0 [0–3]	0.10
AIS rachis	0 [0–2]	0 [0–3]	0.02
Treatments			
Mechanical ventilation	130 (74.2)	33 (45.8)	< 0.001
Acetaminophen	115 (65.3)	44 (61.1)	0.53
Benzodiazepine	109 (61.9)	27 (37.5)	< 0.001
Opioids	144 (81.8)	58 (80.6)	0.82
Neuro-muscular blockers	34 (19.3)	3 (4.2)	0.002
Ketamine	22 (12.5)	2 (2.8)	0.02
Craniectomy	13 (7.4)	0 (0.0)	–
Transfusion	0 [0–2]	0 [0–6]	< 0.001
In-hospital transport	92 (52.3)	43 (59.7)	0.28
Outcome			
28-day mortality	37 (21.0)	8 (11.1)	0.07
Days on mechanical ventilation	7 [4–14]	4 [1–9]	< 0.001
Days on vasopressor infusion	2 [1–4]	2 [1–4]	0.40
Day in the ICU stay	10 [5–20]	8 [4–14]	0.14
Days in the hospital	13 [7–24]	10 [6–15]	0.10
Temperature rhythm			
Period	24 [22–48]	24 [22–46]	0.42
Mesor	37.5 [37.1–37.7]	37.6 [37.2–38.0]	0.01
Amplitude	0.35 [0.27–0.55]	0.34 [0.26–0.54]	0.63

Results are expressed as median [IQR] for quantitative variables and number (%) for categorical variables

*BMI* Body mass index, *SAPS2* Severity Assessment Physiology Score 2, *ISS* Injury Severity Score, *AIS* Abbreviated Injury Scale

**Table 4:** Survival analysis assessing 28-day mortality, patient characteristics, and circadian rhythm parameters of the temperature according to head trauma

	Head trauma				No head trauma			
	Univariate		Multivariate		Univariate		Multivariate	
	HR [95% CI]	*p*	HR [95% CI]	*p*	HR [95% CI]	*p*	HR [95% CI]	*p*
Clinical variables								
Age	1.04 [1.02–1.06]	< 0.001	1.04 [1.02–1.06]	< 0.001	1.07 [1.03–1.12]	< 0.001	1.10 [1.04–1.17]	< 0.001
Sex (male)	1.68 [0.74–3.82]	0.22			2.36 [0.29–19.17]	0.42		
Body mass index	0.91 [0.82–1.01]	0.07			0.90 [0.73–1.10]	0.30		
Glasgow Coma Scale	0.96 [0.87–1.05]	0.33			0.84 [0.75–0.95]	0.004	0.77 (0.65–0.91)	0.003
Intracranial hypertension^a^	7.55 [3.15–18.12]	< 0.001			–	–	–	–
Surgery at admission	0.90 [0.47–1.72]	0.75			0.71 [0.14–3.52]	0.68		
ISS	1.01 [0.98–1.04]	0.47			1.01 [0.97–1.06]	0.57		
Temperature rhythm								
Period	1.00 [0.97–1.02]	0.74			1.05 [0.99–1.11]	0.11		
Mesor	0.41 [0.25–0.65]	< 0.001	0.40 [0.23–0.70]	0.001	0.73 [0.23–2.29]	0.59		
Amplitude	4.48 [1.24–19.20]	0.02	4.73 [1.38–16.22]	0.01	6.10 [1.06–35.25]	0.04	2.94 [0.50–17.25]	0.23

The analyses were performed using Cox regression model to estimate hazard ratios (HR) and their 95% confidence intervals (95% CIs)

^a^Intracranial hypertension not included in the multivariate analysis owing to collinearity with head trauma

## Discussion

Our study aimed at highlighting the circadian clock disruption in trauma patients. We showed that CBT profiles were disrupted early, with alterations in mesor, period, and amplitude. In addition, the changes in CBT variations were associated with the 28-day mortality rate. This finding suggests that the circadian clock may play a major role in the host response to traumatic insults.

Our findings are in line with those of previous studies [28, 29]. In a cohort of 61 ICU patients, Drewry et al. predicted the patients who would develop sepsis up to 72 h before the diagnosis by comparing the temperature profiles of the patients with those of controls [28]. In 22 critically ill patients, Papaioannou et al. used temperature variability to differentiate those with fever due to infection and those with fever due to a non-infectious cause [29]. These data underline the importance of considering temperature variations instead of an isolated single value.

In our study, several variables were associated with CBT variations. BMI was shown to influence CBT during both the perioperative period and ICU stay [30, 31]. Interestingly, higher CBT values were reported in overweight patients. Of note, the overweight ICU patients had a better outcome than those with normal BMI [32]. Low temperature values were previously reported in patients with traumatic brain injury [33, 34]. We previously found a decreased expression of *Per3* in this population [22]. In addition, in our ICU, ketamine was used as a sedative agent in the patients with intracranial hypertension [35]. In rats, ketamine induced dose-dependent hypothermia because of its anti-NMDA effect [36]. This mechanism may explain the association between the decreased CBT amplitude and the administration of ketamine.

The analysis of the period provided surprising results. Indeed, most trauma patients had a period different from the 24-h rhythm. In a cohort of 28 patients without traumatic brain injury, Gazendam et al. showed delayed acrophasis in the most severely ill patients, suggesting a profound alteration of the period associated with critical illness [21]. This is in line with our biological findings showing that acrophase was delayed for most critical mediators. This delay was associated with an increased rate of infection during the ICU stay [22].

Our methodology relied on two mathematical models. First, we performed a Fourier transformation or spectral analysis. For each patient, the spectral analysis identifies the most likely temperature curve period. Then, a Cosinor analysis, based on a sinusoidal approximation of a periodic value, allowed the calculation of three variables, namely, mesor, amplitude, and period. In contrast, Drewry et al. [28] requested visual advice from a group of physicians to identify CBT clock disturbances. Gazendam et al. [21] used a Cosinor function, but the time period was set at 24 h for all patients. Papaioannou et al. [37] analyzed approximate entropy of CBT within 24 h after nosocomial infection diagnosis to predict the mortality rate. The use of different methods complicates the comparisons of the findings of those studies. We made an attempt to approximate the most likely temperature cycle period. However, the Fourier transformation showed that 75 patients had a rhythm period longer than or equal to 48 h. This may reflect a total abolition of circadian rhythm or a longer period that would require several days of analysis.

In our study, advanced age, intracranial hypertension, and early increase in CBT amplitude were independent risk factors for 28-day mortality. Thresholds for mesor (< 36.9 °C) (i.e., the average value of CBT) and amplitude (> 0.6 °C) were associated with poor outcomes. Hypothermia is an independent risk factor for increased 28-day mortality [38, 39]. Amplitude was assessed in small cohorts of patients. In 15 patients, Tweedie et al. [19] showed that the CBT amplitude was > 0.15 °C in non-survivors. In 86 patients with traumatic brain injury, Kirkness et al. reported significantly higher temperature amplitude of 0.1 °C among non-survivors [40]. Conversely, other studies found that a decreased amplitude during the ICU stay was associated with the severity of illness and the mortality rate [41, 42]. However, these data do not contradict our results. Indeed, the logarithmic analysis of the mortality risk as a function of amplitude showed a trend toward excess mortality in the patients with reduced amplitude. In fact, it seems that the 28-day mortality rate was associated with deviation from the normal values of CBT [41–43]. Of note, our findings clearly show significant differences between the head trauma patients and the non-head trauma patients. The effects of CBT disturbances are more pronounced in the head trauma patients.

Our study has several strengths. Our protocol included a large number of patients, as compared to previous studies [21, 28, 37, 42, 43]. Temperature measurements were collected from a central site—this point being the most common limitation encountered in other studies [21, 28, 37, 40, 44]. Bladder temperature monitoring is a validated method for determining CBT—with a minimal error margin and a good correlation with CBT [45]. In addition, this monitoring is simple and inexpensive. Finally, our automatic system of recording at the rate of 1 value per minute resulted in a large amount of data, making our analysis reliable.

This study has some limitations. First, we cannot provide a cause-effect relationship between the different events. Second, owing to our methodology, we were unable to evaluate the effects of the external components such as ambient temperature, physical activity, and feeding [46]. However, in our ICU, as reported above, the room temperature was set by a thermostat. Physical activity and postural changes affect CBT [46], but in the ICU patients, these effects are limited, because 74% of them were on mechanical ventilation. Our patients were bedridden during the study period. Most of the included patients received continuous enteral feeding, erasing the effects of meals between day and night [47]. The light exposure was not recorded, but a local protocol ensured the quality of patients’ sleep with daylight during the day and darkness at night. Third, accidental disconnections of the CBT recording system occurred during the study period. The consequences were limited by the non-inclusion of patients with a lack of data exceeding 6 h. Fourth, few treatments such as fluid expansion were not entered into our statistical analysis although they may play a role in the temperature variations. However, as our results showed, one should note that neither acetaminophen nor transfusion affected the circadian temperature variation. Fifth, because of our system of file analysis, we did not note the occurrence of clinical outcomes such as sepsis or other complications. We considered that the 28-day mortality rate was a relevant and major outcome. Finally, it is a future challenge to transfer our exploratory findings to the bedside. However, in contrast to several studies [22, 48, 49], we show here that the circadian clock alterations occur early in the hospitalization, suggesting that they probably reflect the severity of inflammation. The current evidence does not really support any specific interventions, although guidelines on sedation [50] already considered this concept.

## Conclusions

Our findings underline the early disruption of the circadian clock of CBT after ICU admission for trauma. A level of CBT lower than the normal mesor value and high amplitude were identified as independent risk factors of 28-day mortality in the patients with head trauma. These results are not confirmed in the non-head trauma patients. Further studies will be required to determine the mechanisms and the pathological role of circadian clock disruption during the care of trauma patients.

## Supplementary information

**Additional file 1.** Example of a Fourier transformation followed by a Cosinor analysis to determine rhythmic parameters of the temperature. The Fourier transformation provides a power spectrum (grey lozenges) to determine the most probable rhythm period (red circle=highest power) from 48 h temperature data. Cosinor analysis is a periodic regression method that provides a sinusoidal approximation (dark line) of a rhythm from a set of temperature data (grey dots) with a given period determined with the Fourier transformation. In this example, analysis revealed a period of 22.5 hours, a mesor of 37.4 °C and an amplitude of 0.72 °C.

**Additional file 2.** Definitions and interpretations of circadian parameters.

**Additional file 3.** Characteristic variables of a rhythmic function.

**Additional file 4 **Four examples of central core body temperature rhythm modelling. The dark lines represent the sinusoidal approximation of the Cosinor analysis from the set of temperature data (grey dots). Period, Mesor, and Amplitude of the temperature are respectively for trauma **A**: 6.4 hours, 38.7°C, 0.35°C; trauma **B**: 13.3 hours, 37.8°C, 0.38°C; trauma **C**: 24.0 hours, 37.6°C, 0.30°C; trauma **D**: 41.3 hours, 37.6°C, 0.62 °C.

**Additional file 5.** Individual circadian parameter data after periodic regression.

**Additional file 6.** Factors influencing temperature rhythm parameters (i.e. Period, Mesor, and Amplitude): Univariate logistic regression analysis.

**Additional file 7.** Factors influencing temperature rhythm parameters (i.e. Mesor and Amplitude): Multivariate logistic regression analysis.

## Data Availability

The datasets used and/or analyzed during the current study are available from the corresponding author on reasonable request.

## Abbreviations

BMI: Body mass index
CBT: Core body temperature
CI: Confidence intervals
HR: Hazard ratio
ICU: Intensive care unit
IQR: Interquartile
ISS: Injury Severity Score
OR: Odds ratios
RASS: Richmond Agitation-Sedation Scale
SAPS2: Simplified Acute Physiology Score
SD: Standard deviation
SE: Standard error
