# Supplementary figures and images for: Circadian disruption of core body temperature in trauma patients: a single-center retrospective observational study
Source: J Intensive Care. 2020 Jan 6;8:4. doi: 10.1186/s40560-019-0425-x (PMC6945723; doi:10.1186/s40560-019-0425-x)

## Fourier Transformation

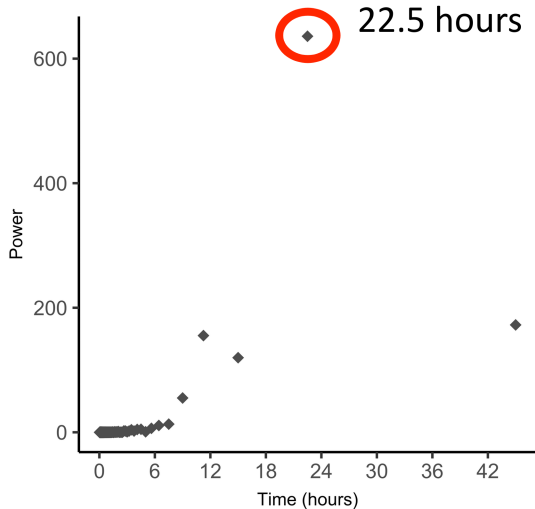

## Cosinor Analysis

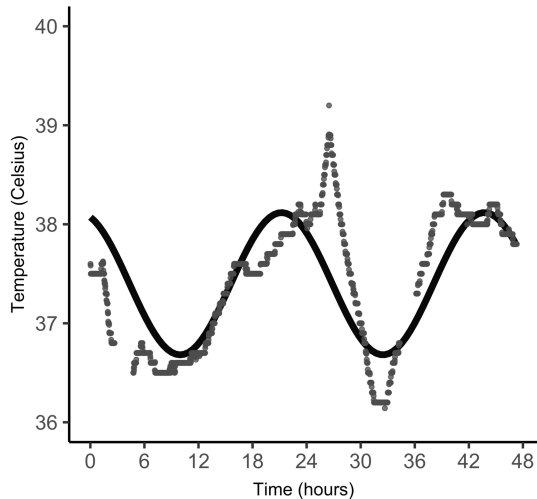

Supplement: Supplementary file 1 — Additional file 1. Example of a Fourier transformation followed by a Cosinor analysis to determine rhythmic parameters of the temperature. The Fourier transformation provides a power spectrum (grey lozenges) to determine the most probable rhythm period (red circle=highest power) from 48 h temperature data. Cosinor analysis is a periodic regression method that provides a sinusoidal approximation (dark line) of a rhythm from a set of temperature data (grey dots) with a given period determined with the Fourier transformation. In this example, analysis revealed a period of 22.5 hours, a mesor of 37.4 °C and an amplitude of 0.72 °C. [file 40560_2019_425_MOESM1_ESM.pdf]

Acrophase

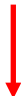

Amplitude

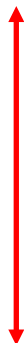

Mesor

Period

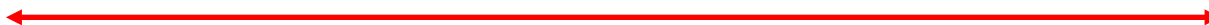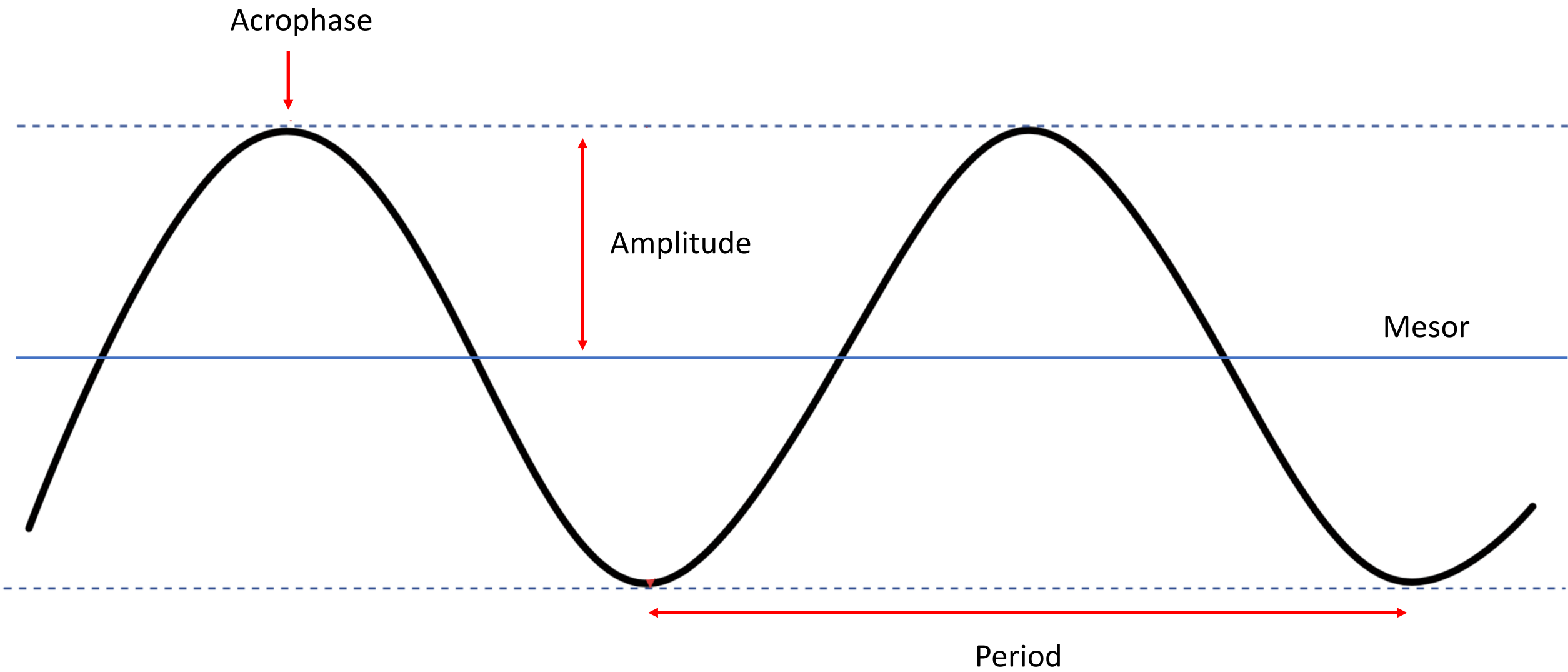

Supplement: Supplementary file 3 — Additional file 3. Characteristic variables of a rhythmic function. [file 40560_2019_425_MOESM3_ESM.pdf]

**A**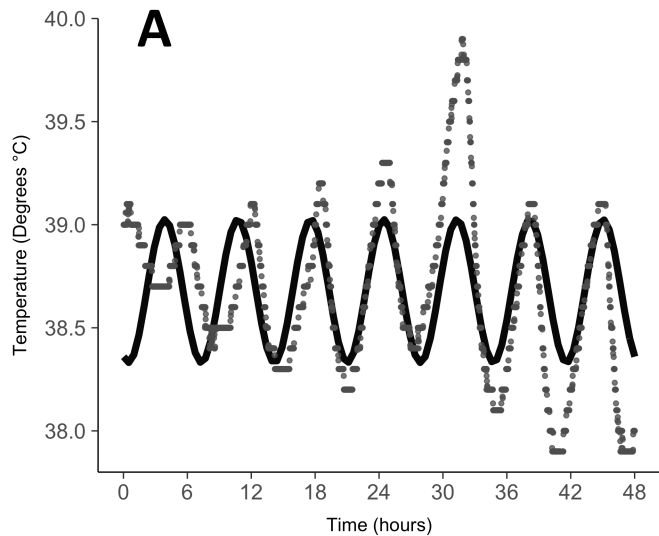**B**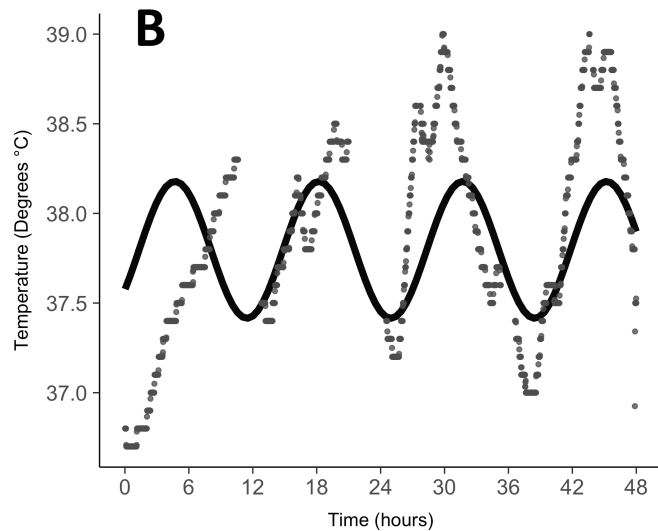**C**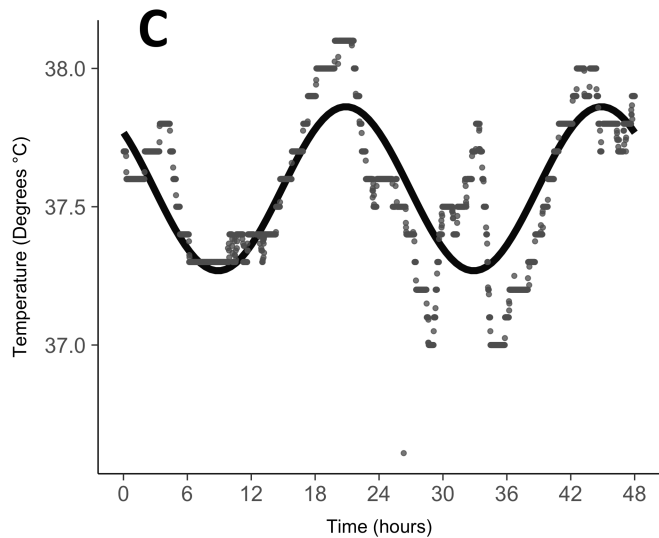**D**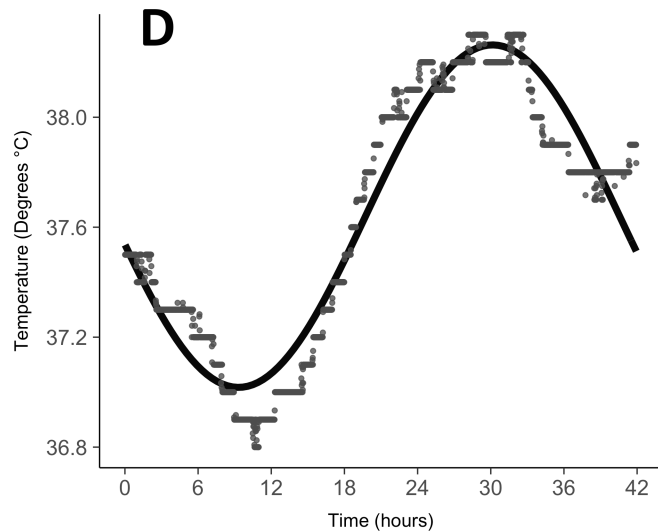

Supplement: Supplementary file 4 — Additional file 4 Four examples of central core body temperature rhythm modelling. The dark lines represent the sinusoidal approximation of the Cosinor analysis from the set of temperature data (grey dots). Period, Mesor, and Amplitude of the temperature are respectively for trauma A: 6.4 hours, 38.7°C, 0.35°C; trauma B: 13.3 hours, 37.8°C, 0.38°C; trauma C: 24.0 hours, 37.6°C, 0.30°C; trauma D: 41.3 hours, 37.6°C, 0.62 °C. [file 40560_2019_425_MOESM4_ESM.pdf]
