# Supplementary material for: Circadian disruption of core body temperature in trauma patients: a single-center retrospective observational study
Source: J Intensive Care. 2020 Jan 6;8:4. doi: 10.1186/s40560-019-0425-x (PMC6945723; doi:10.1186/s40560-019-0425-x)
Supplement: Supplementary file 2 — Additional file 2. Definitions and interpretations of circadian parameters. [file 40560_2019_425_MOESM2_ESM.docx]

**Additional File 2** Definitions and interpretations of circadian parameters

| **Variable** | **Definition** | | **Interpretation** |
| --- | --- | --- | --- |
| MESOR | | MESOR (Midline Estimating Statistic Of Rhythm) represents the mean of the modeled rhythm over the 24-h period | Mean level of concentration of the variable studied |
| Amplitude | | Difference between mesor and the value at the acrophase | Presence of a rhythm if different from zero |
| Period | | Complete cycle time of a variable (about 24 hours for a circadian rhythm) | Decrease or extension of the rhythm period |
| Acrophase | | Time-of-day of the maximum value | Estimation of delayed or advanced rhythm |
