# Supplementary material for: Circadian disruption of core body temperature in trauma patients: a single-center retrospective observational study
Source: J Intensive Care. 2020 Jan 6;8:4. doi: 10.1186/s40560-019-0425-x (PMC6945723; doi:10.1186/s40560-019-0425-x)
Supplement: Supplementary file 6 — Additional file 6. Factors influencing temperature rhythm parameters (i.e. Period, Mesor, and Amplitude): Univariate logistic regression analysis. [file 40560_2019_425_MOESM6_ESM.docx]

**Additional File 6** Factors influencing temperature rhythm parameters (i.e. Period, Mesor, and Amplitude): Univariate logistic regression analysis

|  | **Period** |  |  | **Mesor** |  |  | **Amplitude** |  |  |
| --- | --- | --- | --- | --- | --- | --- | --- | --- | --- |
|  | Coefficient | SE | p | Coefficient | SE | p | Coefficient | SE | p |
| **Demographic** |  |  |  |  |  |  |  |  |  |
| *Age* | 0.037 | 0.048 | 0.44 | 0.001 | 0.002 | 0.78 | -0.001 | 0.001 | 0.38 |
| *Sex (Male)* | -2.213 | 1.992 | 0.27 | 0.161 | 0.084 | 0.06 | 0.048 | 0.035 | 0.17 |
| *Body mass index* | 0.130 | 0.252 | 0.60 | 0.026 | 0.011 | 0.01 | -0.009 | 0.004 | 0.03 |
| **Initial severity** |  |  |  |  |  |  |  |  |  |
| *Glasgow coma scale* | -0.253 | 0.193 | 0.19 | 0.014 | 0.008 | 0.09 | -0.007 | 0.003 | 0.049 |
| *Traumatic brain injury* | 1.082 | 1.952 | 0.58 | -0.232 | 0.081 | 0.005 | 0.001 | 0.034 | 0.99 |
| *Intracranial hypertension* | -0.882 | 1.896 | 0.64 | -0.339 | 0.077 | <0.01 | 0.083 | 0.033 | 0.01 |
| *Surgery at admission* | -1.688 | 1.789 | 0.35 | -0.033 | 0.076 | 0.66 | -0.034 | 0.031 | 0.28 |
| *SAPS2* | 0.034 | 0.053 | 0.52 | -0.003 | 0.002 | 0.13 | 0.003 | 0.001 | 0.005 |
| *ISS* | -0.002 | 0.070 | 0.98 | -0.006 | 0.003 | 0.04 | 0.003 | 0.001 | 0.007 |
| *AIS head or neck* | -0.099 | 0.405 | 0.81 | -0.038 | 0.017 | 0.03 | -0.001 | 0.007 | 0.96 |
| *AIS chest* | -0.335 | 0.495 | 0.50 | -0.006 | 0.021 | 0.79 | 0.004 | 0.009 | 0.63 |
| *AIS abdomen or pelvic* | -0.004 | 0.531 | 0.99 | 0.007 | 0.022 | 0.74 | -0.001 | 0.009 | 0.91 |
| *AIS extremities* | 0.264 | 0.593 | 0.66 | -0.020 | 0.025 | 0.42 | 0.004 | 0.010 | 0.68 |
| *AIS rachis* | 0.484 | 0.562 | 0.39 | -0.008 | 0.024 | 0.75 | 0.013 | 0.010 | 0.18 |
| **Treatments** |  |  |  |  |  |  |  |  |  |
| *Mechanical ventilation* | 0.595 | 2.026 | 0.77 | 0.162 | 0.085 | 0.06 | 0.102 | 0.035 | 0.004 |
| *Acetaminophen* | -0.221 | 1.848 | 0.90 | -0.021 | 0.078 | 0.79 | 0.027 | 0.032 | 0.40 |
| *Benzodiazepine* | 2.887 | 1.781 | 0.62 | 0.119 | 0.075 | 0.11 | 0.127 | 0.030 | <0.01 |
| *Opioids* | 0.947 | 2.280 | 0.68 | -0.098 | 0.096 | 0.31 | 0.092 | 0.040 | 0.02 |
| *Neuromuscular blockers* | -0.401 | 2.488 | 0.87 | -0.518 | 0.100 | <0.001 | 0.133 | 0.043 | 0.002 |
| *Ketamine* | -0.689 | 2.997 | 0.82 | -0.628 | 0.121 | <0.001 | 0.250 | 0.050 | <0.001 |
| *Craniectomy* | -2.886 | 3.974 | 0.47 | 0.201 | 0.268 | 0.23 | 0.158 | 0.069 | 0.002 |
| *Transfusion* | 0.012 | 0.255 | 0.96 | 0.009 | 0.011 | 0.38 | 0.001 | 0.005 | 0.93 |
| **In-hospital transport** | -2.168 | 1.775 | 0.22 | -0.032 | 0.075 | 0.68 | -0.058 | 0.031 | 0.06 |

*SE* standard error, *SAPS2* simplified acute physiology score 2
