# Supplementary material for: Circadian disruption of core body temperature in trauma patients: a single-center retrospective observational study
Source: J Intensive Care. 2020 Jan 6;8:4. doi: 10.1186/s40560-019-0425-x (PMC6945723; doi:10.1186/s40560-019-0425-x)
Supplement: Supplementary file 7 — Additional file 7. Factors influencing temperature rhythm parameters (i.e. Mesor and Amplitude): Multivariate logistic regression analysis. [file 40560_2019_425_MOESM7_ESM.docx]

**Additional File 7** Factors influencing temperature rhythm parameters (i.e. Mesor and Amplitude): Multivariate logistic regression analysis

|  |  |  | **Mesor** |  |  |  | **Amplitude** |  |
| --- | --- | --- | --- | --- | --- | --- | --- | --- |
| Demographic | Coefficient | SE | OR [95% CI] | p | Coefficient | SE | OR [95% CI] | p |
| Body mass index | 0.021 | 0.010 | 1.02 [1.00-1.04] | 0.03 | -0.008 | 0.004 | 0.99 [0.98-1.00] | 0.07 |
| Initial severity |  |  |  |  |  |  |  |  |
| Intracranial hypertension^a^ | -0.160 | 0.084 | 0.85 [0.72-1.01] | 0.06 | -0.004 | 0.037 | 0.99 [0.93-1.07] | 0.92 |
| ISS^b^ | -0.003 | 0.003 | 0.99 [0.99-1.00] | 0.26 | 0.002 | 0.001 | 1.00 [0.99-1.00] | 0.09 |
| Treatments |  |  |  |  |  |  |  |  |
| Mechanical ventilation^c^ | - | - | - | - | 0.059 | 0.037 | 1.06 [0.99-1.14] | 0.12 |
| Neuromuscular blockers | -0.235 | 0.125 | 0.79 [0.62-1.01] | 0.06 | -0.014 | -0.053 | 0.99 [0.89-1.09] | 0.79 |
| Ketamine | -0.331 | 0.150 | 0.72 [0.53-0.96] | 0.03 | 0.235 | 0.063 | 1.26 [1.12-1.43] | <0.001 |

*SAPS2* simplified acute physiology score 2, SE standard error, OR Odds Ratio, CI: confidence interval

^a^Glasgow coma scale score, Traumatic brain injury and craniectomy not included in the multivariate analysis owing to collinearity with intracranial hypertension

^b^SAPS2 not included in the multivariate analysis owing to collinearity with ISS

^c^Benzodiazepine and opioids not included in the multivariate analysis owing to collinearity with mechanical ventilation
